# Quantifying Forearm Muscle Activity during Wrist and Finger Movements by Means of Multi-Channel Electromyography

**DOI:** 10.1371/journal.pone.0109943

**Published:** 2014-10-07

**Authors:** Marco Gazzoni, Nicolò Celadon, Davide Mastrapasqua, Marco Paleari, Valentina Margaria, Paolo Ariano

**Affiliations:** 1 LISiN, Department of Electronics and Telecommunications, Politecnico di Torino, Torino, Italy; 2 Center for Space Human Robotics, Istituto Italiano di Tecnologia, Torino, Italy; University of Minnesota Medical School, United States of America

## Abstract

The study of hand and finger movement is an important topic with applications in prosthetics, rehabilitation, and ergonomics. Surface electromyography (sEMG) is the gold standard for the analysis of muscle activation. Previous studies investigated the optimal electrode number and positioning on the forearm to obtain information representative of muscle activation and robust to movements. However, the sEMG spatial distribution on the forearm during hand and finger movements and its changes due to different hand positions has never been quantified. The aim of this work is to quantify 1) the spatial localization of surface EMG activity of distinct forearm muscles during dynamic free movements of wrist and single fingers and 2) the effect of hand position on sEMG activity distribution. The subjects performed cyclic dynamic tasks involving the wrist and the fingers. The wrist tasks and the hand opening/closing task were performed with the hand in prone and neutral positions. A sensorized glove was used for kinematics recording. sEMG signals were acquired from the forearm muscles using a grid of 112 electrodes integrated into a stretchable textile sleeve. The areas of sEMG activity have been identified by a segmentation technique after a data dimensionality reduction step based on Non Negative Matrix Factorization applied to the EMG envelopes. The results show that 1) it is possible to identify distinct areas of sEMG activity on the forearm for different fingers; 2) hand position influences sEMG activity level and spatial distribution. This work gives new quantitative information about sEMG activity distribution on the forearm in healthy subjects and provides a basis for future works on the identification of optimal electrode configuration for sEMG based control of prostheses, exoskeletons, or orthoses. An example of use of this information for the optimization of the detection system for the estimation of joint kinematics from sEMG is reported.

## Introduction

The human hand shows highly complex motor skills, which are essential for many daily activities. The study of hand and finger movement is an important topic with potential applications in prosthetics, rehabilitation, and ergonomics [1] [2]. Electromyography (EMG) is the gold standard for the detection and analysis of muscle activation. Surface EMG (sEMG) signal detected with up to eight bipolar detection systems is commonly used to control multifunction prosthesis [1] [3] [4] [5] [6]. However, the recent development of sophisticated hand prostheses mimicking the high number of degrees of freedom (DOF) of the human wrist/hand complex push forward the development of more advanced control systems. The use of multi-channel detection systems has been recently proposed in order to increase the informative content of the detected sEMG [7] [8] [9] [10] [11]. Mono or bi-dimensional electrode arrays increase the reliability and the information content of sEMG and provide information not obtainable through traditional detection systems. On the other hand, signals detected with multi-channel systems show a certain degree of redundancy and, in practical application, the number of electrodes must be limited as much as possible [12].

It has been proven that the classification accuracy of movements based on sEMG improves by increasing the number of electrodes, up to a limit beyond which a plateau is reached [13] [14]. Many works in literature evaluate the effect of 1) the electrode number and positioning and 2) the arm positions on the classification accuracy of movements using an exhaustive approach and simulating electrode shift.

The aim of this work was to quantify, by means of a sEMG multi-channel detection system, 1) whether it is possible to spatially localize the sEMG activity related to the activation of distinct forearm muscles during dynamic free movements of the wrist and single fingers, and 2) the effect of hand position (prone vs. neutral) on the sEMG activity in terms of changes in amplitude and spatial distribution. The study provides quantitative knowledge about sEMG activity distribution on the forearm as a basis for future works on the identification of optimal electrode number and positioning for sEMG based control of prostheses, exoskeletons, or orthoses. Preliminary tests about the use of the obtained knowledge in the optimization of the detection system for the estimation of joint kinematics from sEMG are shown.

## Materials and Methods

### Subjects and experimental procedures

**Figure 1:**
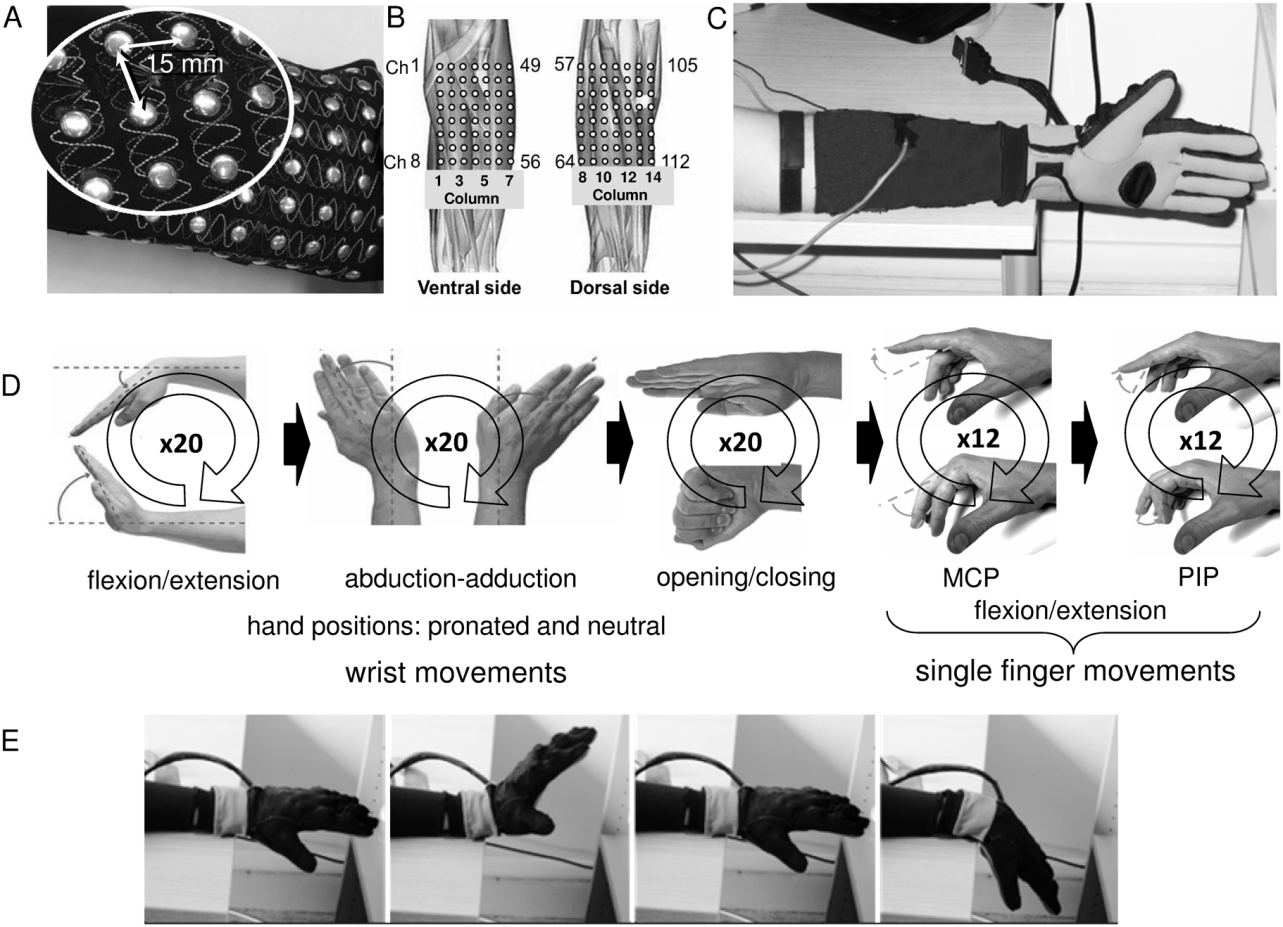
Experimental setup and protocol. (A) Wearable detection system consisting of a grid of 112 silver circular electrodes (14×8, diameter: 6 mm, inter-electrode distance: 15 mm) integrated into a stretchable textile sleeve with the 14 columns of electrodes placed around the forearm circumference. (B) Approximate position of the electrode matrix on the forearm. (C) Sensorized hand and forearm. The subjects had worn the sEMG textile detection system with the first column of electrodes in correspondence of the ulna and with the more proximal electrodes at approximately 2 cm from the elbow crease. A sensorized hand glove was used to record the kinematics of the hand and of the fingers. (D) The protocol consisted in 12 different cyclic dynamic tasks involving the wrist and index, middle, ring, and little fingers (see text for details). E) One example of wrist flexion/extension with the hand in prone position.

Eight male healthy subjects, aged between 25 and 40 years, participated in the study. All subjects provided written informed consent and the protocol was within a program of research approved by the local Regional Ethics Committee (Commissione di Vigilanza, Servizio Sanitario Nazionale – Regione Piemonte – ASL 1– Torino, Italy). The subjects were seated in a height adjustable chair in a comfortable position with the shoulder abducted and flexed ∼30°, the elbow flexed ∼120° and with the elbow and the forearm resting on a horizontal surface. The subjects were instructed to perform cyclic dynamic single DOF tasks involving the wrist and the fingers (Table 1). The wrist tasks consisted in the flexion/extension and adduction/abduction with the hand in two positions (prone and neutral) (Figure 1d). The tasks for each single finger consisted in the flexion/extension of the metacarpophalangeal (MCP) and of the proximal interphalangeal (PIP) joint performed with the hand in neutral position (Figure 1d). For each task, the subject was instructed to start from the rest position, to reach and maintain for 2 s the first target position (maximum flexion or adduction), to reach and maintain for 2 s the second target position (maximum extension or abduction), and then to return to the rest position. The movement speed was selected in order to maximize the finger movement independence and to obtain a consistent movement pace [15] [16] [17]. Each task was repeated cyclically (20 times for the wrist movements and 12 times for the finger movements) (Table 1). No feedback was provided to the subjects to regulate the target positions, but the validation of the motions was performed offline on the basis of kinematic data recorded with a sensorized glove (see section *C. Hand kinematics recordings*).

**Table 1:** Movements Included In The Protocol.

task id	Segment	Joint	Movement	Position	N. rep.
1			flex/ext	neutral	
2	Hand	Wrist		prone	
3			add/abd	neutral	20
4				prone	
5	All fingers	MCP^1^, PIP^2^, and DIP^3^		neural	
6				prone	
7	Index				
8	Middle	MCP^1^			
9	Ring				
10	Little		flex/ext	neutral	12
11	Index				
12	Middle	PIP^2^			
13	Ring				
14	little				

The Subjects Were Asked To Perform 14 Tasks, Tasks 1–4 Involving The Wrist Joint With The Hand In Two Positions (Neutral And Prone), Tasks (5–14) Involving The Fingers. ^1^MCP: Metacarpophalangeal, ^2^PIP: Proximal Interphalangeal, And ^3^DIP: Distal Interphalangeal.

### Surface electromyography recordings

sEMG signals have been acquired from the left forearm muscles using a wearable detection system consisting of a grid of 112 silver circular electrodes (14×8, diameter: 6 mm, inter-electrode distance (IED): 15 mm LISiN unpublished internal report) integrated into a stretchable textile sleeve (medium size, forearm circumference: 24–28 cm) with the 14 columns of electrodes placed around the forearm circumference (Figure 1A). Before the subject put on the sleeve, the forearm skin was lightly wetted. The sleeve was worn by the subject with the first column of electrodes in correspondence of the ulna and with the more proximal electrodes at approximately 2 cm from the elbow crease (Figure 1B). When the sleeve was worn by the subject, the center to center distance between two adjacent electrodes slightly varied depending on the circumference of the forearm (forearm circumference (mean±std): 26.5±1.3 cm). The sEMG signals were recorded with a monopolar configuration with the reference electrode placed on the wrist, amplified with a gain of 500 (EMG-USB128, LISiN – OT Bioelettronica, Torino, Italy), band-pass filtered (3-dB bandwidth: 10–750 Hz), sampled at 2048 samples/s per channel, and digitally converted (12 bit A/D converter). The amplifier was connected via USB to a PC for data storage.

### Hand kinematics recordings

The sensorized hand glove HumanGlove (Humanware s.r.l., Pisa, Italy) was used to record the kinematics of the hand and of the fingers. HumanGlove is a wireless sensing glove designed to measure the hand’s posture using twenty-two Hall Effect sensors. A glove calibration procedure was performed for each subject using the Graphical Virtual Hand (GVH) calibration software provided by Humanware. Kinematic data acquisition has been performed through the GVH software (sampling frequency: 50 samples/s per channel, 12 bit A/D converter) storing the angle values for each joint together with the sampling time. The joint angle signals from index finger MCP and wrist flexion/extension were acquired also through two auxiliary inputs of the EMG-USB system in order to perform the off-line synchronization of sEMG and kinematics signals.

### Signal processing

The sEMG and kinematics signals were processed offline with MATLAB R2010b (The Mathworks Inc., Natick, MA, USA).

#### Kinematic signal analysis

**Figure 2:**
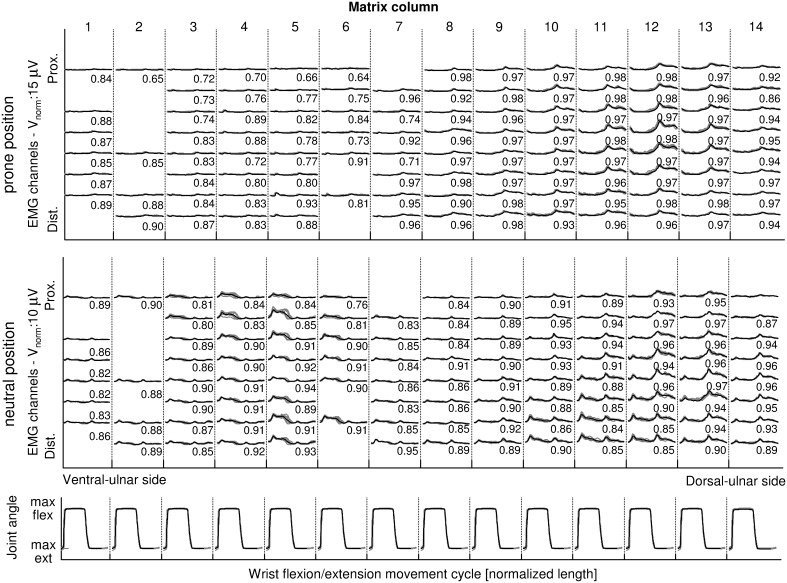
Example of the sEMG envelopes during a wrist flexion/extension task. The envelopes are shown for wrist flexion/extension with the hand in prone position (top) and in neutral position (middle). For each condition, the envelopes estimated for each movement cycle are shown superimposed (gray lines). The columns correspond to the electrode matrix columns (medio-lateral direction) while the rows corresponds to the rows of the electrode matrix (proximal-distal direction). On the bottom, in correspondence of each sEMG column, the wrist flexion/extension angle time courses for all movement cycles are represented superimposed. The black line represents the mean envelope/joint angle. The number near to each sEMG envelope is the CMC value calculated on all cycles. The envelopes show a good repeatability with CMC higher than 0.8 except for the bad channels. The missing channels are bad channels.

Kinematic signals were re-sampled to 2048 Hz and synchronized with sEMG through the common synchronization signals. For each task, the angular signal detected from the joint involved in the movement was used to identify the movement cycles. A movement cycle was defined between the beginnings of two subsequent flexion/adduction dynamic phases (Figure 2). The beginning of each flexion/adduction dynamic phase was determined as the time when the joint angle exceeded 2° the value corresponding to the maximum extension/abduction reached during the previous isometric phase (Figure 2). Cycles identified as outliers on the basis of the range of motion were excluded in the following analysis.

#### sEMG signal analysis

The monopolar sEMG signals were digitally filtered with a 4^th^ order Butterworth non-causal filter (20–450 Hz) and power line interference (50 Hz and its four higher harmonics) was removed using spectral interpolation [19]. sEMG channels corresponding to electrodes with bad electrode-skin contact were identified using the outlier identification method proposed by Marateb et al. [20]. sEMG envelopes were calculated by rectifying and then low-pass filtering the sEMG signals using a bidirectional 4^th^ order Butterworth filter with 1 Hz cutoff frequency.

The envelopes of sEMG epochs corresponding to each movement cycle were extracted and time-normalized in order to obtain 500 data points for each cycle. The repeatability of the sEMG envelopes among the movement cycles was evaluated using the Coefficient of Multiple Correlation (CMC) for all electrodes and all tasks. The mean sEMG envelope was calculated for each subject, task, and channel by averaging the sEMG envelopes on all cycles.

a) Identification of sEMG clusters. Non Negative Matrix Factorization (NNMF) has been used as a dimensionality reduction pre-processing step for clustering. NNMF decomposes a non-negative matrix 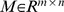 into two non-negative factors 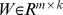 and 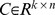 (where 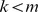), that is 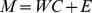 where 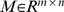 represents the multi-channel measured data (*m* channels, *n* samples), 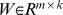 contains the NMF basis (each column of W is a basis vector of length *m*, that represent invariant patterns of activity across the channels), 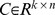 represents the associated coefficients (the columns contain the weights of each basis for each channel: each element c_ij_, specifies the contributions of each component *j* to the measured activation pattern *M_i_*), 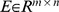 is the error [21] [22] [23]. The matrices W and C are estimated by minimizing the Euclidean Distance between *M* and *WC*.

In the present study, the NNMF basis and the associated coefficients were extracted, for each subject, by applying the NNMF algorithm to the concatenation of the sEMG mean envelopes of all channels and tasks. To avoid local minima, the NNMF algorithm was repeated 100 times for each subject. The result showing the lowest squared error between original and reconstructed sEMG envelopes was kept.

The number *N* of modules needed for the approximation of the sEMG envelope was assessed by repeating, for all subjects, the NNMF analysis with the number of modules *N* varying between 1 and 10. The quality of envelope reconstruction with *N* modules was quantified by means of the variation accounted for (VAF) defined as the variation that can be explained by the model: VAF = 1–SSE/SST, where SSE (sum of squared errors) is the unexplained variation and SST (total sum of squares of *M*) is the pooled variation of the data [22]. The least number of modules that accounted for 90% of VAF was selected [22].

The segmentation algorithm proposed by Vieira et al [24] was applied to the *C* maps of coefficients to identify the electrode clusters where each module was mostly represented. The algorithm consists in two steps: the first step identifies the areas with different amplitude by applying watershed segmentation to the equalized amplitude *C* map. In the second step, the electrodes whose coefficients are higher than a threshold (70% of the maximum value in the cluster) are considered as belonging to a cluster of activity.

b) Comparison of sEMG activity areas for different movements and conditions. In order to study the effect of different hand positions (prone or neutral) on the sEMG activity distribution the centre of gravity (COG) of electrode clusters was estimated. The COG’s coordinates for each electrode cluster were defined as:
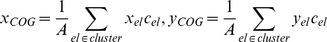
where *x_el_* is the electrode position in ulnar–radial direction (*x_el_* 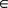 [1 14]), *y_el_* the electrode position in proximal–distal direction (*y*_el_ 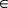 [1 8]), *c_el_* the weight associated to the electrode *el,* and *A* the sum of the weights over all the electrodes belonging to the cluster.

The degree of overlapping of the sEMG areas identified for tasks involving different fingers or joints was calculated as the intersection of the clusters (that is the number of electrodes common to each pair of clusters). This number was normalized to 1) the number of channels in the smallest of the two clusters to obtain the degree of inclusion of the smallest area into the biggest one and 2) the number of channels in the largest of the two clusters to obtain a measure of the area not common to both clusters.

### Joint angle estimation from monopolar sEMG envelopes

In order to test the importance of identifying specific areas of sEMG activity during hand and finger movements, preliminary tests were performed comparing the performance in the reconstruction of joint angles from sEMG of two sets of electrodes: a) one ring of 14 electrodes placed around the forearm 2 cm from the elbow crease and b) a set of electrodes whose position has been selected corresponding to the barycenter of the identified areas of activity.

In order to reconstruct the joint angles from monopolar sEMG envelopes, a set of feed-forward multilayer perceptrons (MLPs), one for each joint, were used [25]. Each MLP had one hidden layer of six neurons connected to the input layer through a tan-sigmoid transfer function and to the output layer through a linear function [25]. The MLPs were trained using the Levenberg-Marquardt back propagation algorithm. 70% and 30% of the number of task cycles were used for the training and validation sets respectively, according to the four-fold cross validation procedure. The performance of joint reconstruction was evaluated using the coefficient of determination *r^2^*.

### Statistics

The Wilcoxon test was used to study the effect of the hand position on the COG coordinates with statistics significance level set to 0.05. Analysis of variance (ANOVA) followed by the post-hoc Student Newman-Kelus test was applied to investigate the effect of channel selection on the joint angle reconstruction quality (*r^2^*).

## Results

All subjects were able to perform the complete protocol. The wearable textile detection system showed, after the first two test sessions, 8 electrodes (mostly located on the first column positioned near the ulna) with broken connection. On an average, 2–3 additional electrodes per subject were discarded because of bad electrode-skin contact.

Kinematic signals showed a good repeatability among movement cycles for all subjects and all movements. For three subjects a percentage of cycles reported in the following (median (IQR 25%–75%)) was excluded from the analysis: subject AlFa (2.5% (0.0–33.3)), subject PaMa (8.6% (0–20%)), and subject RoSt: (5.5% (0–30%)).

Figure 2 shows one example of sEMG envelopes during wrist adduction/abduction task for one subject. CMC values are also reported. Median CMC values calculated on the sEMG envelopes were always higher than 0.90 for the wrist movements and higher than 0.80 for finger movements.

### Identification of clusters of sEMG activity

**Figure 3:**
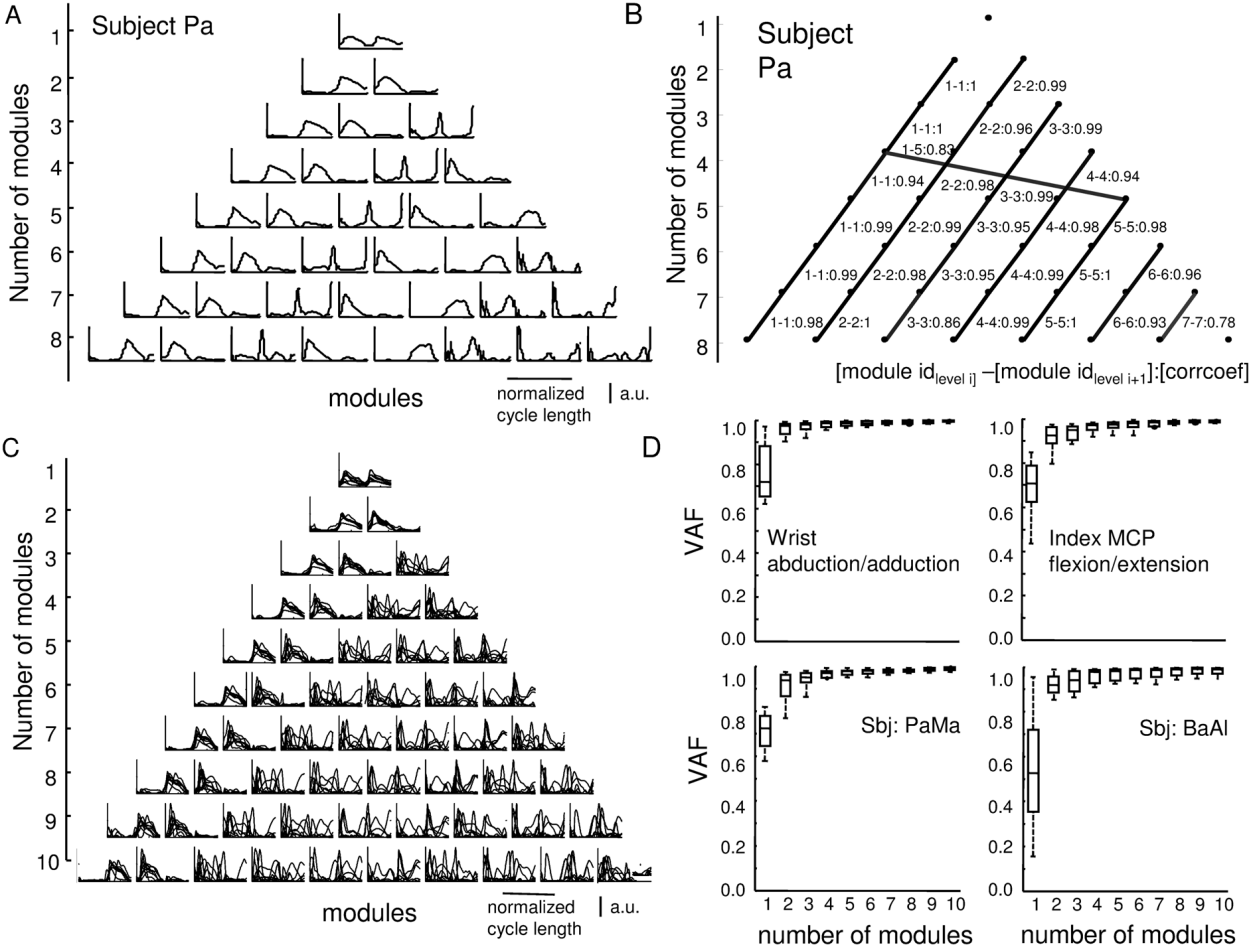
Motor modules extracted from sEMG envelopes using NNMF analysis for one representative subject. (A) Example of the motor modules extracted for the subject Pa (first 8 sets of modules). The modules identified in the set with N modules are essentially preserved in the set with N+1 modules. (B) Similarities between sets with different number of modules. The nodes of each row of the triangle represent the modules extracted from sets with a number of modules ranging from 1 to 8. The links between the nodes in two adjacent rows connect modules that are similar (correlation coefficient above 0.7). The degree of similarity is indicated by the thickness of the link and the value shown close to each link. (C) Superposition of the motor modules identified for all subjects. The first two modules are very similar for all subjects while differences among the subjects are represented starting from the third module. (D) Top: variance accounted for (VAF) as a function of the number of motor modules for two representative tasks (wrist flexion/extension and ring MCP flexion/extension). Bottom: VAF as a function of the number of motor modules for two representative subjects. Three motor modules allow an accurate approximation of the sEMG envelopes (median VAF>0.9).

Figure 3A shows, for a representative subject, the motor modules extracted from sEMG envelops using NNMF. Increasing the number of modules from 2 to 10, the set with *N* modules is essentially preserved in the set with *N*+1 modules. Figure 3B shows one example of the analysis of the similarities among the basis functions in the sets with *N* and *N*+1 modules. Motor module 1 is primarily active during the second half of the movement cycle; it mainly represents extensors activity during extension/flexion tasks and abductor activity during abduction/adduction tasks. Motor module 2 is active during the first half of the movement cycle; it mainly represents the extensor activity during extension/flexion tasks and adductor activity during abduction/adduction tasks. Figure 3C shows, overlapped, the sets of motor modules (from 1 to 8) identified for all subjects.

The dimensionality analysis determined that three motor modules are sufficient to reconstruct the sEMG envelopes with VAF>0.9. Figure 3D shows VAF values as a function of the number of modules for two movements and for two subjects.

**Figure 4:**
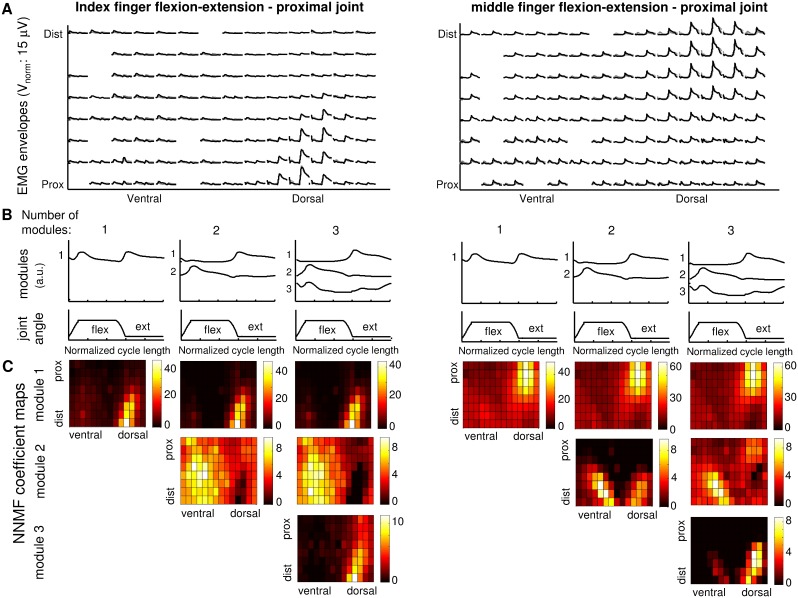
Example of modules and weights resulting from the NMF analysis for flexion/extension of the index (on the left) and middle (on the right) fingers for one representative subject. (A) sEMG mean envelopes estimated on all movement cycles for each channel (black line) and their approximation obtained with three NNMF modules (gray line). Two distinct areas of activity for index and middle fingers can be identified. (B) The three sets of modules identified for this subject varying the number of modules from one to three. (C) NNMF coefficient maps for the identified modules. It is possible to observe that increasing the number of modules from one to two, it is possible to separate and highlight the areas of activity during extension (map of coefficient for module 1) and flexion (map of coefficient for module 2). By increasing the number of modules from two to three, the maps corresponding to the first two modules remain mainly unchanged while module 3 highlights some detail of sEMG activity distribution.

Figure 4 shows one representative example of the weights associated to each motor module and channel for the index flexion/extension (left) and middle flexion/extension (right). Increasing the number of modules from one to two, it is possible to separate the areas of activity during extension (map of coefficient for module 1) and flexion (map of coefficient for module 2). By increasing the number of modules from two to three, the maps corresponding to the first two modules remain mainly unchanged while module 3 highlights some detail of sEMG activity distribution. While in the case of index finger module 3 is mainly represented in the same area than module 1 (with lower weights), in the case of middle finger, the area where module 3 is mainly represented corresponds to the area of activity identified for the index finger extension (module 1). In this case module 3 highlights a small activity of index extensor needed to counteract the biomechanical coupling between the tow fingers and then to avoid the index flexion during middle flexion.

#### Distance and overlapping of the electrode areas identified for single finger movements

**Figure 5:**
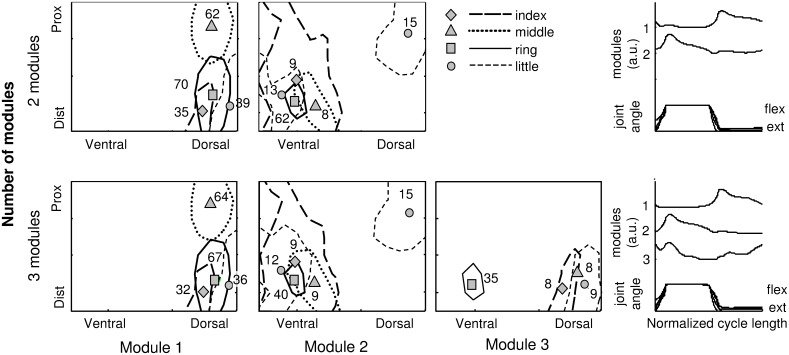
Example of the areas of activity detected during isolated flexion/extension movement of all fingers (index, ring, middle, and little). Top: areas identified using the set with two NNMF modules. Bottom: areas identified using the set with three NNMF modules. each area the position of the barycenter and the weight of the NNMF module in that position are reported. The results obtained with both sets of modules are very similar and will be discussed together. For all fingers, it is possible to identify partially not-superimposed areas of activity with respect to other fingers suggesting the possibility to distinguish single finger movements on the basis of sEMG activity distribution.

**Figure 6:**
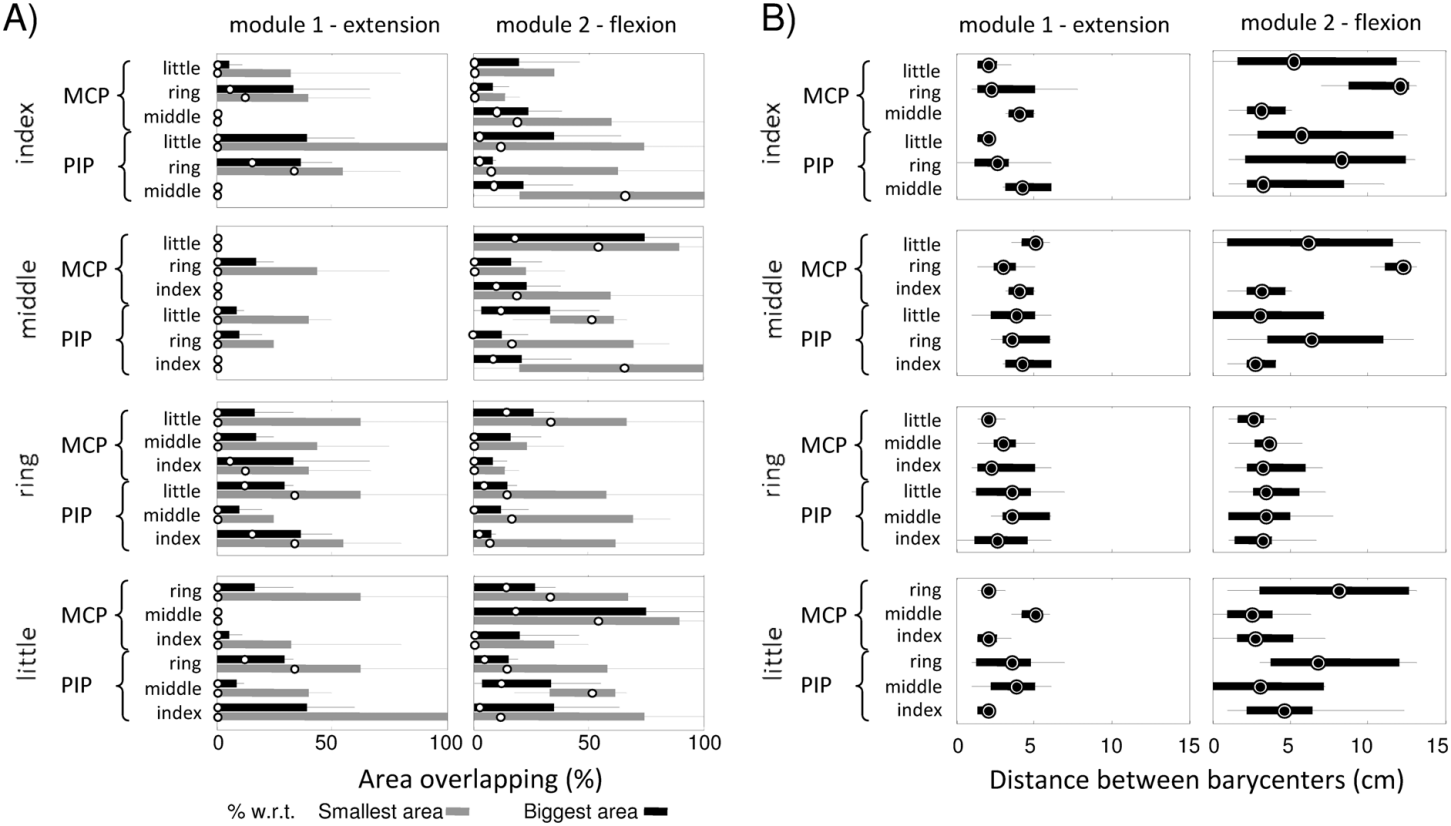
Percentage of overlapping and distance among the barycenters for the identified areas of sEMG activity. In A) the percentage of overlapping between the area of sEMG activity identified for each finger with respect to the areas of sEMG activity identified for each one of the other fingers are shown. The percentage of overlapping is calculated with respect to the number of electrodes in both the smallest (gray) and largest (black) activity area. In B) the distance between the barycenters of the areas of sEMG activity, for all possible pairs of fingers and all modules, are shown. The values (median (25%–75%)) are reported for MCP and PIP joints.

Figure 5 shows a representative example of the sEMG activity areas identified for the single fingers using two and three modules. The positions of the barycenters and the corresponding NNMF weights are also shown. The results obtained with both sets of modules are very similar and will be discussed together. For all fingers, it is possible to identify partially not-superimposed areas of activity with respect to other fingers suggesting the possibility to distinguish single finger movements on the basis of sEMG activity distribution. Figure 6 shows, for all possible pairs of fingers: A) the percentage of overlapping of sEMG activity areas for all possible pairs of fingers (with respect to both the smallest and the largest area) for flexion and extension movements and B) the distance between the barycenters of these areas.

From Figure 5 is it possible to observe, for a specific subject, the middle finger (dotted line and triangle symbol) is the finger with the more separated area of activity during extension; Figure 6A confirms the result for all subjects: the median value of the percentage of overlapping for the middle finger with respect to each other finger is 0% for both normalizations and Figure 6B shows the distance between the barycenter of the activity area of the middle finger with respect to the position of the barycenters of all other fingers is higher than 2*IED.

On the other hand, Figure 5 shows the ring finger (solid line and square symbol) is the only finger whose area of activity during extension intersects with the activity areas of all other fingers and Figure 6A confirms the results for all subjects. However, the percentage of superposition normalized with respect to the biggest area is always lower than 50% and Figure 6B shows the distance between the barycenter of the activity area of the ring finger with respect to the position of the barycenters of all other fingers is always greater than one IED. These results highlight the possibility of identifying a specific sub-area of activity for all fingers.

#### Displacement of the electrode areas between prone and supine hand positions

**Figure 7:**
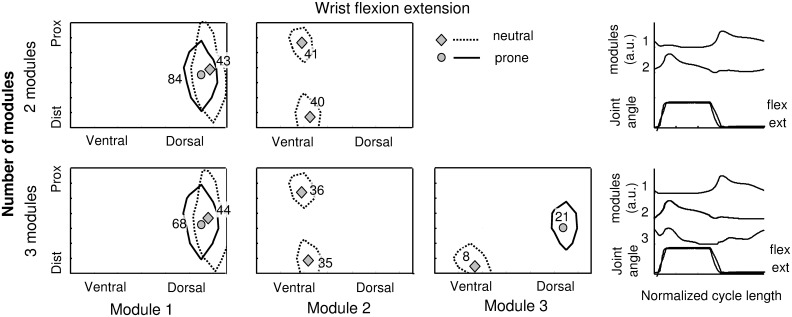
Example of sEMG areas of activity detected during the wrist flexion/extension task with the hand in neutral and prone position. Top: areas of sEMG activity identified using the set with two NNMF modules. Bottom: areas of sEMG activities identified using the set with three NNMF modules. For each area the position of the barycenter and the weight of the NNMF modules in that position are reported.

Figure 7 shows one representative example of the areas of activity identified during wrist flexion/extension with the hand in prone and neutral positions. For module 1 the overlapping of the areas identified for the two hand positions is high with a shift toward the right moving from prone to neutral position. The weight associated to module 1 is higher in prone than in neutral position (84 vs 43 for the analysis with 2 modules and 68 vs 44 for the analysis with 3 modules). Module 2 is present only in the case of neutral position. These results are in accordance with the fact that in the prone position the subject has to move the hand counter-gravity during the extension and with the favor of gravity during flexion while in neutral position gravity has the same effect for both movements. The Wilcoxon test disclosed a statistical difference (p<0.01) in the position of COG in ulnar-radial direction between the neutral and prone hand positions while no differences were detected along the proximal–distal direction. The distance between the COG in the two hand postures along the ulnar-radial direction was (median (25%–75%)): 0.8 IED (0.7–1.1) for wrist flexion/extension, 1.2 IED (0.1–1.4) for wrist adduction/abduction, and 1.0 IED (0.5–1.6) for hand opening/closing. Table 2 shows the barycenter weight ratio between prone and neutral position for each module. For flexion/extension movement it is possible to observe a ratio higher than 1 for module 1 (associated with the extensor muscle activity) and lower than 1 for module 2 (associated with flexor muscles). These values are in agreement with the effect of gravity on the movement that facilitates flexion and opposes to extension in prone position. For adduction/abduction movement it is possible to observe a ratio lower than 1 for module 1 (associated with the abductor muscle activity) and higher than 1 for module 2 (associated with adductor muscles). Also in this case the values are in agreement with the effect of gravity on the movement.

**Table 2:** Ratio between the module weights Associated To the area barycenterS in prone versus neutral position.

		Weight ratio (prone/neutral)median (25%–75%)		
Segment	Movement	Module		
		1	2	3
**hand**	flex/ext	1.4 (1.3–1.7)	0.5 (0.3–0.8)	0.8 (0.4–2.0)
	add/abd	0.7 (0.6–1.1)	1.9 (0.9–3.5)	0.7 (0.4–1.2)
**all fingers**	flex/ext	0.9 (0.5–1.4)	1.1 (0.8–1.6)	1.0 (0.7–1.4)

### Joint angle estimation from monopolar sEMG envelopes

Statistical analysis was performed on the quality of reconstruction of joint angles from sEMG detected using two different sets of electrodes: a) one ring of 14 electrodes equally spaced and positioned 2 cm from the elbow crease and b) one set of electrodes whose position has been optimally selected in correspondence of the barycenter of the identified areas of sEMG for each finger. Results showed the quality of joint angle reconstruction depends on the electrode set (*r^2^_ring_*: (69±12%); *r^2^_COG_*: (80±6%); p<0.01) and the best reconstruction was obtained with the electrode set positioned in correspondence of the barycenters of the identified sEMG activity areas.

## Discussion

In this work we have investigated, by means of a wearable multi-channel sEMG detection system, 1) whether it is possible to spatially identify distinct areas of sEMG activity in the forearm during dynamic free movements of the wrist and single fingers, 2) the effect of the hand position on the sEMG activity. The results show that specific distinguishable areas of activity can be identified during wrist and single finger movements suggesting the use of targeted positioning of electrodes could improve the performances of sEMG-based movement classification systems. Considering the main application could be in prosthetics, these results must be validated in amputees.

### Textile detection systems

Most studies on hand prosthesis myoelectric control make use of eight or less pairs of standard electrodes [26] [27] placed either with reference to particular muscles or equidistant over an area of interest. In the last years, prosthetic hands with high dexterity have been developed and this highlighted the need for complex control systems. In order to address this issue, recently some studies investigated the use of multi-channel sEMG [9] [10] [28]. The multi-channel sEMG recording provides useful information to improve the recognition of fine movements. On the other hand it requires a complex and cumbersome setup with the need to place many electrodes on the forearm and to manage many connectors [10] [29]. In order to simplify the electrode setup Farina et al. used a textile sleeve with four grids of 5×5 electrodes to detect sEMG signals from upper arm and forearm for the recognition of wrist and forearm movements [30]. In this work a textile stretchable sleeve with 112 electrodes has been used to detect sEMG from a large area of the forearm. This technology allows a simplified positioning of the sEMG detection system and a significant reduction of the setup time without the need to target specific muscles. Moreover, it reduces the problems due to the cumbersome of multi-channel detection systems used in literature. The loss of information about the sEMG activity in correspondence of the 8 electrodes, whose connection broken during the experimental protocol, does not affect the results because mainly located near the ulna.

### Identification of sEMG activity areas

In literature, some studies focused on the effect of the number of electrodes on the movement classification accuracy for prosthesis control and on the identification of the minimum number of sEMG electrodes that allow maintaining good classification performances. The general approach is based on the evaluation of the performances of a movement classifier changing the number and set of electrodes in an iterative way. Liu and Zhou [10] applied a straightforward sequential feedforward selection algorithm, which iteratively added the most informative channels, as determined by empirical classification performance. The results showed that it was feasible to reduce the number of sEMG channels (down to 8) while maintaining high (>95%) classification accuracy. In a similar way, Li et al. applied a straightforward exhaustive search algorithm [31]. Daley et al. [28] showed that, in healthy subjects, a ring of eight channels produced significantly lower classification performance with respect to a subset of electrodes each one optimally positioned longitudinally and transversally on the forearm. This result suggests careful electrode placement is needed to recognize fine motor tasks. Other studies reported an improvement in classification accuracy with increasing the number of electrodes up to a threshold, after which the performances start to decrease [13] [34].

In this study, a different approach has been adopted; the NNMF technique has been applied to sEMG signals recorded using a multi-channel detection system to identify the clusters of sEMG activity during different wrist and finger movements. Multi-channel high density sEMG provides detailed topographical maps of sEMG activity and allows to verify if distinguishable sEMG spatio-temporal patterns are produced during different tasks and to identify the electrode positions detecting the higher sEMG activity. The use of NNMF for linear factorization of forearm sEMG signals even without targeting specific muscles for electrode positioning has been shown to be appropriate in a recent work [18].

The dimensionality analysis on NNMF results showed that three modules account for 90% of VAF and the modules identified in the set with *N* modules are essentially preserved in the set with *N*+1 modules. This result shows that the choice of the number of modules is not critical and the analysis performed with a particular number of modules will be consistent with the analysis based on larger sets.

The first two modules extracted with NNMF show a high level of similarity among all subjects (Figure 3). Module 1 mainly represents extensor/abductor muscle activity; it is active during the extension/abduction phase of the movement cycle with the peak of activity occurring at the end of the extension/abduction dynamic phase. Module 2 mainly represents flexors/adductor muscle activity; it is mainly active during the flexion/adduction phase of the movement cycle with the peak of activity occurring at the end of the flexion/adduction dynamic phase. Module 3 is subject specific; it represents some details of the time course of sEMG envelope but the areas where it is mainly represented are included in the areas where the first two modules are already represented so it introduces limited new subject-specific information about sEMG activity distribution on the forearm. For these reasons, in the following analysis, the first two modules have been considered.

### Identification of specific sEMG areas for single fingers

The hand shows substantial mechanical coupling across adjacent fingers [7] [32] [33] [34]; fully independent movements of the fingers are not possible and the activation of antagonist muscles is required to limit the movement of the other fingers. For this reason, during the single finger movements, the subject was asked to move the target finger with no constraints about the movement of the other fingers to avoid the activation of the antagonist muscles.

Some works in literature indicate that sEMG cannot be used to distinguish single finger muscle activity because of high levels of crosstalk [35] [36]. However, other researchers showed that the decoding of individual and combined finger movements and the control of a prosthesis with complex movement dexterity can be obtained by sEMG [26] [37]. Leijnse demonstrated, with anatomic dissections, that the ED parts to the different fingers show constant and widely spaced anatomical locations that promote independent function [38]. These findings suggested the hypothesis that single finger sEMG activity assessment should be possible and this has been demonstrated using small (4 mm) bipolar surface electrodes appropriately placed [25].

The results of this work about single finger sEMG activity, showed that the use of multi-channel detection systems allows to identify distinct areas of sEMG activity in the forearm for different fingers, in accordance with recent findings [25] [38].

The results of the comparison of sEMG areas detected during movement involving PIP and MCP joints show a high superposition of the activity areas and a distance between the barycenters of the activity areas lower than one IED. This is in accordance with Kamper et al. [39] that showed extrinsic flexor muscles initiate MCP flexion and produce simultaneous motion at the MCP, PIP, and DIP joints.

### Effect of rotation of the hand on sEMG activity distribution

In most studies for sEMG-based multifunctional prosthesis control, the tests are performed with the subject in a reference position and the training and testing sEMG signals are recorded in a constant position of the arm. However, in daily living activities the user’s arm position changes and different forearm muscle activities could be observed during the same movement. The changes in sEMG activity distribution can be due to two main factors: 1) the electrode displacement due to skin and muscle shift and 2) the different effect of gravity on body segments.

The works published in literature evaluate the effect of different arm positions on the classification accuracy or simulate the shift of the electrode. Fougler et al. [40] showed that changes in arm position during the use of a myoelectric prosthesis could substantially impair the sEMG-based movement recognition with an increase of average classification error from 3.8% to 18%. Geng et al. [41] showed that the impact of arm position on sEMG pattern-recognition performances is a little stronger in intact arm than in amputated arm. Boschmann and Platzner [29] showed that, to compensate an electrode displacement of 1 cm on the classification accuracy, the number of detection points must be increased from 8 to 32. Hargrove et al [42] studied the effect of 1 cm electrode displacement in longitudinal and/or transversal direction on pattern classification accuracy using an IED of 3 cm, and proposed a classifier training strategy to accommodate performance degradation.

In this work the use of multi-channel sEMG allowed to investigate the changes in sEMG activity distribution during the same movement when hand position changes (neutral and prone). The results showed a shift of the COG of the sEMG activity areas of approximately 1 IED for all considered wrist movements. Moreover, we investigated if sEMG activity level was influenced by the hand position. The ratio between the NNMF weights associated with each module for all tasks in prone versus neutral position highlight the influence of the hand position on the muscle activation. For flexion/extension and adduction/abduction movements the ratios reflect the influence of gravity that facilitates flexion and opposes to extension in prone position and facilitates abduction and opposes adduction in neutral position. These results must be carefully considered in the development of sEMG-based control for orthoses or prostheses. Forearm pronation-supination is, in fact, one of the most common movements carried out in conjunction with grasps and hand movements.

### Limitations of the study

The main limitation of the present study, considering one of the primary applications could be the control of prosthesis, is the tested subjects are all healthy subjects while amputees may have significantly different muscle maps than able-bodied individuals. However, this work serves as a methodological foundation for future work. The feasibility and real advantages of the targeted selection of electrode positioning with respect to an electrode ring shown in this work must be verified in amputees. Moreover, this work could contribute in the development of orthoses and new human-computer interfaces.

In this work the sEMG signals were recorded in monopolar modality and are probably e affected by crosstalk. However, due to the close interlacing of the narrow extensor muscles [38], spatial crosstalk filtering [43] [44] cannot be applied. Moreover, Daley et al. [9] showed no differences in classification accuracy of hand movements using monopolar or single differential detection in normal subjects while monopolar recordings showed slightly higher classification accuracies in amputees. The issue requires further investigation of the crosstalk problem.

Only a male population has been studied because of the availability of only one size (medium) of the prototype of sleeve for sEMG detection. However, the uniform distribution of the electrodes around the forearm circumference, allows sampling the sEMG activity in the same relative positions regardless of the forearm circumference. For this reason the obtained results are expected to be generalizable to a population with smaller or bigger forearm.

## Conclusions

The textile sleeve used in this work provided a fast setup and a reliable detection of multi-channel sEMG signals from forearm muscles during dynamic tasks. The effect of hand position on the distribution and on the level of sEMG activity during wrist and hand movements has been quantified. The results give new information, not available in literature, that are independent from the characteristics and robustness of a specific classification system and provides a basis for a future work on the identification of optimal electrode number and positioning for sEMG based prosthesis control.
